# Supplementary material for: Association between oral microbiome and seven types of cancers in East Asian population: a two-sample Mendelian randomization analysis
Source: Front Mol Biosci. 2023 Nov 21;10:1327893. doi: 10.3389/fmolb.2023.1327893 (PMC10702768; doi:10.3389/fmolb.2023.1327893)
Supplement: Supplementary file 1 [file DataSheet1.ZIP › Supplementary Materials/MR plots for tongue/tongue═╝/Pancreatic cancer/╥╚╧┘░⌐_╔╕╤í╜ß╣√/pheno.1497_to_pancreatic cancer_leave_one_out.pdf]

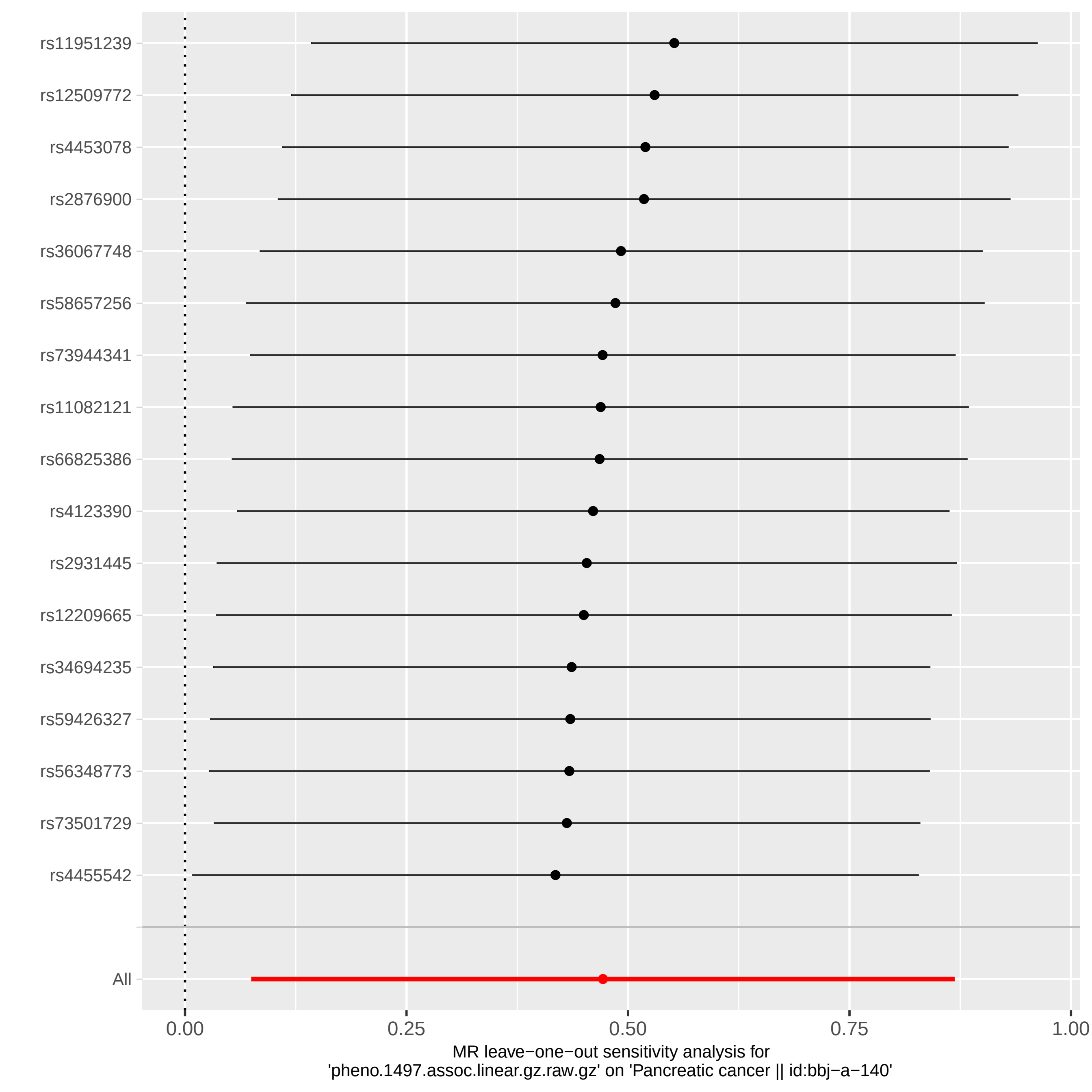
rs11951239
rs12509772
rs4453078
rs2876900
rs36067748
rs58657256
rs73944341
rs11082121
rs66825386
rs4123390
rs2931445
rs12209665
rs34694235
rs59426327
rs56348773
rs73501729
rs4455542
All
0.00
0.25
0.50
0.75
1.00
MR leave−one−out sensitivity analysis for
'pheno.1497.assoc.linear.gz.raw.gz' on 'Pancreatic cancer || id:bbj−a−140'
